# Evolutionary History and Phylogeography of Rabies Viruses Associated with Outbreaks in Trinidad

**DOI:** 10.1371/journal.pntd.0002365

**Published:** 2013-08-22

**Authors:** Janine F. R. Seetahal, Andres Velasco-Villa, Orchid M. Allicock, Abiodun A. Adesiyun, Joseph Bissessar, Kirk Amour, Annmarie Phillip-Hosein, Denise A. Marston, Lorraine M. McElhinney, Mang Shi, Cheryl-Ann Wharwood, Anthony R. Fooks, Christine V. F. Carrington

**Affiliations:** 1 Department of Preclinical Sciences, Faculty of Medical Sciences, The University of the West Indies, St. Augustine, Trinidad and Tobago; 2 Veterinary Diagnostic Laboratory, Ministry of Food Production, Champs Fleurs, Trinidad and Tobago; 3 Rabies Program, Centers for Disease Control and Prevention, Atlanta, Georgia, United States of America; 4 School of Veterinary Medicine, Faculty of Medical Sciences, The University of the West Indies, St. Augustine, Trinidad and Tobago; 5 National Animal Disease Centre, Centeno, Trinidad and Tobago; 6 Penal Demonstration Station, Penal, Trinidad and Tobago; 7 Wildlife Zoonoses and Vector-Borne Diseases Research Group, Animal Health and Veterinary Laboratories Agency (AHVLA), Addlestone, Surrey, United Kingdom; Universite de la Mediterranee, France

## Abstract

Bat rabies is an emerging disease of public health significance in the Americas. The Caribbean island of Trinidad experiences periodic outbreaks within the livestock population. We performed molecular characterisation of Trinidad rabies virus (RABV) and used a Bayesian phylogeographic approach to investigate the extent to which outbreaks are a result of *in situ* evolution versus importation of virus from the nearby South American mainland. Trinidadian RABV sequences were confirmed as bat variant and clustered with *Desmodus rotundus* (vampire bat) related sequences. They fell into two largely temporally defined lineages designated Trinidad I and II. The Trinidad I lineage which included sequences from 1997–2000 (all but two of which were from the northeast of the island) was most closely related to RABV from Ecuador (2005, 2007), French Guiana (1990) and Venezuela (1993, 1994). Trinidad II comprised sequences from the southwest of the island, which clustered into two groups: Trinidad IIa, which included one sequence each from 2000 and 2007, and Trinidad IIb including all 2010 sequences. The Trinidad II sequences were most closely related to sequences from Brazil (1999, 2004) and Uruguay (2007, 2008). Phylogeographic analyses support three separate RABV introductions from the mainland from which each of the three Trinidadian lineages arose. The estimated dates for the introductions and subsequent lineage expansions suggest periods of *in situ* evolution within Trinidad following each introduction. These data also indicate co-circulation of Trinidad lineage I and IIa during 2000. In light of these findings and the likely vampire bat origin of Trinidadian RABV, further studies should be conducted to investigate the relationship between RABV spatiotemporal dynamics and vampire bat population ecology, in particular any movement between the mainland and Trinidad.

## Author Summary

The Caribbean island of Trinidad experiences periodic rabies virus (RABV) outbreaks within the livestock population. In this study, we inferred the evolutionary history of RABV in the Americas and reconstructed past patterns of RABV geographic spread in order to address the question of whether Trinidadian outbreaks arise from locally maintained RABV or are the result of virus importation from the mainland (presumably via infected bats). Our results provide statistical support for three importation events that gave rise to each of three Trinidadian vampire bat-associated lineages identified in the study. They also indicate limited periods of *in situ* evolution within Trinidad following each of these introductions. The results also support Mexico and Brazil as major epicenters for the expansion of RABV associated with vampire bats throughout the Americas and consequently to Trinidad. The findings of our study are particularly relevant to local RABV monitoring and control. In addition to justifying vampire bats as the main target for active rabies surveillance and control activities in Trinidad, they suggest that more intense surveillance of regions that lie close to the mainland may be warranted. Finally, in light of these findings, further studies should be conducted to investigate the relationship between RABV spatiotemporal dynamics and vampire bat population ecology.

## Introduction

Rabies has been a well-known disease since ancient times, and is thought to be the inspiration for mythical and superstitious beliefs in numerous cultures [1], [2]. It is historically one of the most significant zoonotic diseases, a consequence of its near 100% case fatality rate and ubiquitous global distribution [3], [4]. Today, rabies is considered a neglected disease and is of major public health importance worldwide as both an emerging and re-emerging disease [2], [3], [5]. The etiological agent is a single stranded negative-sense RNA virus belonging to the family *Rhabdoviridae*, genus *Lyssavirus* which consists of 12 species that are largely assigned into two distinct phylogroups [6]–[8] with two further viruses awaiting classification [9]. Rabies virus (RABV) belongs to phylogroup I and is maintained as an enzootic agent in several mammalian species within the orders Carnivora and Chiroptera with reservoir host species differing among geographic regions [2], [10]. RABV perpetuated and transmitted by terrestrial mammals (Carnivora) have a global circulation and are phylogenetically distinct from those transmitted by bats (Chiroptera), which are restricted to the Americas [6], [11]. In the Americas, where RABV is the only known lyssavirus species [12], [13], based on molecular and antigenic typing techniques bat-transmitted variants cluster into several distinct bat species-associated lineages [3], [10], [11], [14], [15]–[17].

Rabies has been documented throughout the Americas including several Caribbean islands [18], but the island of Trinidad (which lies about 7 miles off the northeastern coast of South America) is the only Caribbean island with vampire bat-transmitted rabies [19]. The link between bats and human paralytic rabies was established in 1931 in Trinidad [20], [21] during a historic multi-species rabies epidemic (1925–1937). This epidemic recorded 73 human cases and the loss of thousands of livestock animals [19], [22], [23]. Although no human rabies cases associated with bats have been reported since 1937 [19], [24], and the last reported case of canine transmitted human rabies occurred in 1912 [24], there have been periodic rabies epizootics in the livestock population [19], [24], [25], with the most recent significant epizootic event occurring in 2010 [25].

Wright et al [26] previously suggested that RABV outbreaks in Trinidad originate from Venezuela. This was based on phylogenetic analysis of nucleoprotein (N) gene fragments from six Trinidadian RABV isolates of bovine origin which identified two distinct lineages of bat RABV, both belonging to the South American antigenic variant three (AVG 3) [27]. While this scenario is plausible given the proximity of Trinidad to Venezuela, the small sample size, limited time frame covered and the basic phylogenetic analysis performed (UPGMA trees) [26] do not allow robust conclusions to be drawn. In the current study we have used a statistically robust Bayesian coalescent approach to investigate the evolutionary dynamics of RABV in Trinidad and to reveal the pattern of RABV gene flow between Trinidad and mainland South America. Analyses were based on a data set of 183 partial N gene sequences from the Americas, 37 of which were derived from equine, caprine, ovine and bovine rabies cases in Trinidad between 1997 and 2010.

## Methods

### Source of viruses

**Figure 1:**
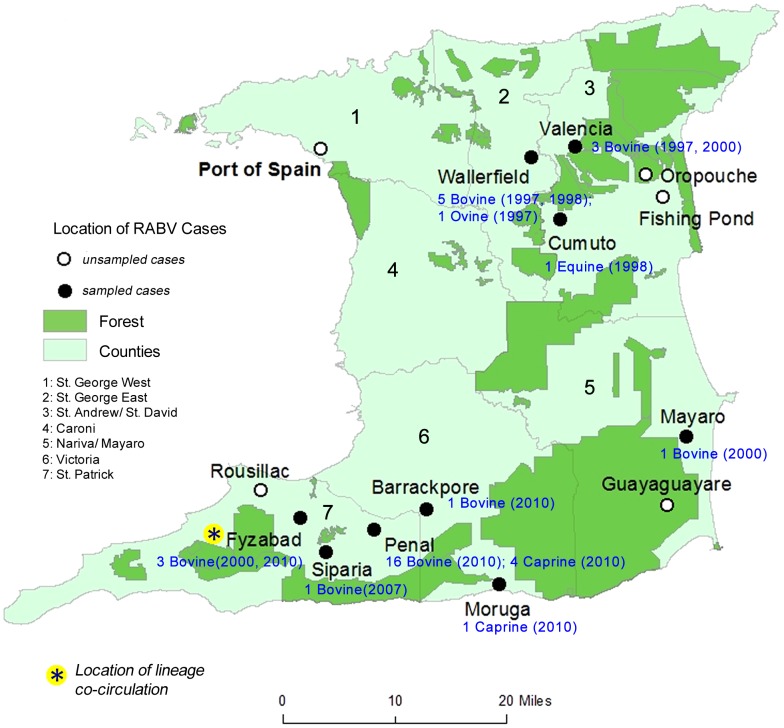
Geographical locations of confirmed RABV cases in Trinidad during the period 1997 to 2010. Locations of cases are indicated by circles, with black circles indicating locations from which RABV sequences were derived for the current study and white circles designating unsampled locations.. For sampled locations, the number of cases from which sequences were derived and their date and species of origin are indicated in blue. The location of co-circulation of Trinidad I and Trinidad IIa lineages is also indicated by an asterisk.

The thirty-seven (37) Trinidad RABV viruses were derived from brain tissue collected from RABV positive livestock (bovine, caprine, ovine and equine) between 1997 and 2010 under the national rabies surveillance program. All brain samples were confirmed positive at the Veterinary Diagnostic Laboratory (VDL) of the Trinidad and Tobago Ministry of Food Production, using the Direct Fluorescent Antibody (DFA) test, in accordance with the protocol recommended by the Centers for Disease Control (CDC), Atlanta, Georgia [28]. Figure 1 illustrates the geographical distribution of the Trinidad RABV samples used in the present study and together with Table 1 gives details on the species source and year of isolation. Figure S1 illustrates the total annual number of laboratory confirmed animal rabies cases for the period under study (1997–2010) [25].

**Table 1:** Trinidadian sequences used for phylogenetic analysis identified by geographical, temporal and species origin.

			Location			
Isolate	Year	Source	Area	County	Accession Number	Trinidad Lineage
2010_50_CAP_PE_TRIN	2010	Caprine	Penal	St. Patrick	KF413598	II b
2010_54_CAP_PE_TRIN	2010	Caprine	Penal	St. Patrick	KF413582	II b
2010_56_BOV_PE_TRIN	2010	Bovine	Penal	St. Patrick	KF413595	II b
2010_15_BOV_PE_TRIN	2010	Bovine	Penal	St. Patrick	KF413597	II b
2010_26_BOV_PE_TRIN	2010	Bovine	Penal	St. Patrick	KF413614	II b
2010_53_BOV_PE_TRIN	2010	Bovine	Penal	St. Patrick	KF413594	II b
2010_25_BOV_PE_TRIN	2010	Bovine	Penal	St. Patrick	KF413612	II b
2010_51_CAP_PE_TRIN	2010	Caprine	Penal	St. Patrick	KF413581	II b
2010_20_CAP_MO_TRIN	2010	Caprine	Moruga	Victoria	KF413609	II b
2010_19_CAP_PE_TRIN	2010	Caprine	Penal	St. Patrick	KF413613	II b
2010_18_BOV_BP_TRIN	2010	Bovine	Barrackpore	Victoria	KF413591	II b
2010_16_BOV_PE_TRIN	2010	Bovine	Penal	St. Patrick	KF413603	II b
2010_22_BOV_PE_TRIN	2010	Bovine	Penal	St. Patrick	KF413586	II b
2010_28_BOV_PE_TRIN	2010	Bovine	Penal	St. Patrick	KF413607	II b
2010_52_BOV_PE_TRIN	2010	Bovine	Penal	St. Patrick	KF413585	II b
2010_39_BOV_PE_TRIN	2010	Bovine	Penal	St. Patrick	KF413583	II b
2010_35_BOV_PE_TRIN	2010	Bovine	Penal	St. Patrick	KF413580	II b
2010_27_BOV_PE_TRIN	2010	Bovine	Penal	St. Patrick	KF413604	II b
2010_23_BOV_FY_TRIN	2010	Bovine	Fyzabad	St. Patrick	KF413578	II b
2010_32_BOV_PE_TRIN	2010	Bovine	Penal	St. Patrick	KF413601	II b
2010_38_BOV_PE_TRIN	2010	Bovine	Penal	St. Patrick	KF413587	II b
2010_40_BOV_PE_TRIN	2010	Bovine	Penal	St. Patrick	KF413592	II b
2010_48_BOV_PE_TRIN	2010	Bovine	Penal	St. Patrick	KF413579	II b
2007_14_BOV_SP_TRIN	2007	Bovine	Siparia	St. Patrick	KF413606	II a
2000_9_BOV_FY_TRIN	2000	Bovine	Fyzabad	St. Patrick	KF413599	II a
2000_BOV_FY_TRIN¥	2000	Bovine	Fyzabad	St. Patrick	KF413588	I
2000_11_BOV_MY_TRIN	2000	Bovine	Mayaro	Nariva/Mayaro	KF413589	I
2000_BOV_VA_TRIN¥	2000	Bovine	Valencia	St. Andrew/St.David	KF413590	I
2000_10_BOV_VA_TRIN	2000	Bovine	Valencia	St. Andrew/St.David	KF413610	I
1998_7_BOV_WF_TRIN	1998	Bovine	Wallerfield	St. George East	KF413600	I
1998_6_EQ_CM_TRIN	1998	Equine	Cumuto	St. Andrew/St.David	KF413584	I
1997_OVI_WF_TRIN¥	1997	Ovine	Wallerfield	St. George East	KF413608	I
1997_45_BOV_WF_TRIN	1997	Bovine	Wallerfield	St. George East	KF413593	I
1997_BOV_VA_TRIN¥	1997	Bovine	Valencia	St. Andrew/St.David	KF413602	I
1997_44_BOV_WF_TRIN	1997	Bovine	Wallerfield	St. George East	KF413611	I
1997_41_BOV_WF_TRIN	1997	Bovine	Wallerfield	St. George East	KF413605	I
1997_3_BOV_WF_TRIN	1997	Bovine	Wallerfield	St. George East	KF413596	I

¥ Sequences obtained from the repository of the Rabies Program, CDC and from the Wildlife Zoonoses and Vector-Borne Diseases Research Group, Animal Health and Veterinary Laboratories Agency (Weybridge).

### RNA extraction, cDNA synthesis and amplification of N gene fragments

For each sample, 50 mg of the RABV positive brain tissue was manually homogenized with 100 µl of molecular grade water and total RNA extracted using Trizol according to the manufacturer's instructions (Invitrogen). RNA was then stored at −70°C until further use. A 542 bp portion of the nucleoprotein (N) gene was amplified by RT-PCR using the degenerate forward primer N921 (5′-YGTGTTCAAYCTHATYCACTT-3′) at position 991–1011 and non-degenerate reverse primer 304 (5′-TTGACGAAGATCTTGCTCAT- 3′) at position 1514–1533 [29] both positions according to the full genome sequence of the fixed rabies virus strain, SAD B19 [30]. For each sample 10 µl of RNA was added to 2 µl of the primer dilutions (5 µM) and centrifuged. Samples were then denatured at 94°C for 1 minute, cooled on ice (∼3 minutes) and 14 µl of reverse transcription reaction (RTRX) buffer, containing reverse transcriptase and protector RNase inhibitor, was added before incubation at 42°C for 90 minutes (with 4°C hold). For PCR, 80 µl of PCR buffer solution, containing Amplitaq and primer, was added to each RT reaction tube, briefly centrifuged and placed in a preheated (94°C) thermocycler. Thermocycling was performed at 94°C for 1 minute followed by 40 cycles of PCR (94°C for 30 seconds, 37°C for 30 seconds, 72°C for 90 seconds with a 7 minute extension at 72°C). Amplicons were visualized on 1.5% agarose containing ethidium bromide (EtBr).

### Nucleotide sequencing

PCR amplicons were excised from the agarose gel and purified using the “ExoSAP-IT purification system” (USB Corporation Cleveland Ohio, USA), before being subjected to direct sequencing using the BigDye Terminator v1.1 cycle sequencing kit (Roche) and an Applied Biosystems 3100 Genetic Analyzer. For each sample 5 to 10 µL of purified amplicon were placed into two separate PCR tubes for forward and reverse cycle sequencing reactions. Two microliters of 3.2 µM forward (N921) and reverse (304) primers and 4 µl of Big Dye Terminator v1.1 were added and the mixture subjected to 25 cycles of PCR at 96°C, 10 seconds; 50°C, for 5 seconds; and 60°C for 4 minutes.

### Nucleotide sequence data set

The data set (n = 183) comprised 33 Trinidadian sequences derived during the study together with four sequences obtained from the CDC, Rabies Program Repository and the Wildlife Zoonoses and Vector-borne Diseases Research Group, AHVLA (Weybridge, UK), and previously published RABV nucleoprotein gene sequences from South and Central America (i.e. Peru, Brazil, Mexico, Columbia, Argentina, Uruguay, El Salvador, Ecuador, French Guiana, Uruguay, Honduras, and Venezuela) downloaded from GenBank [31]. Only Genbank submissions that included both a date and location of origin were considered. Table S1 shows details of all sequences used in the study including accession numbers, dates, species and countries of origin.

Sequences were manually aligned using ClustalX version 2.1 [32] then visually inspected and edited using Bioedit version 7.1.3 [33] before being trimmed to a common length of 363 nt.

### Phylogenetic analysis

Phylogenetic analysis was performed on the aforementioned data set in order to investigate the evolutionary relationships and patterns of RABV gene flow between Trinidad and the South and Central American mainland. This was done using BEAST (v1.6.1) which uses a Bayesian Monte Carlo Markov Chain (MCMC) method to jointly estimate substitution rates, divergence times and demographic histories of the sampled lineages [34], [35]. Analysis was performed under the best fit-nucleotide substitution model, which was identified as the Transition Model plus Gamma (TN+Γ_4_) using FindModel [36]. A relaxed uncorrelated lognormal clock and the Bayesian skyline plot (BSP) coalescent model [37] which does not does not assume any particular demographic scenario *a priori* were chosen. To test the hypothesis that RABV is periodically imported into Trinidad from the South American mainland, a phylogeographic analysis was conducted, with a reversible discrete diffusion model described by Lemey et al [35] also implemented in BEAST. This diffusion model uses the countries of the sampled isolates to reconstruct the ancestral location states of the internal nodes from the posterior time-scaled tree distribution.

Adequate sampling of the model's parameters was achieved by running four MCMC analyses for 100 million generations each, sampling every 10,000 states. LogCombiner (http://tree. bio.ed.ac.uk/software) was then used to combine runs after removing 10% burn-in (such that ESS values were >200). The BEAST output was summarized using Tracer (available at http://tree.bio.ed.ac.uk/software/tracer/) and then visualized using the FigTree (v1.3.1) software package (available at http://tree.bio.ed.ac.uk/software/figtree/) [34], [35].

## Results

### Phylogenetic analysis

**Figure 2:**
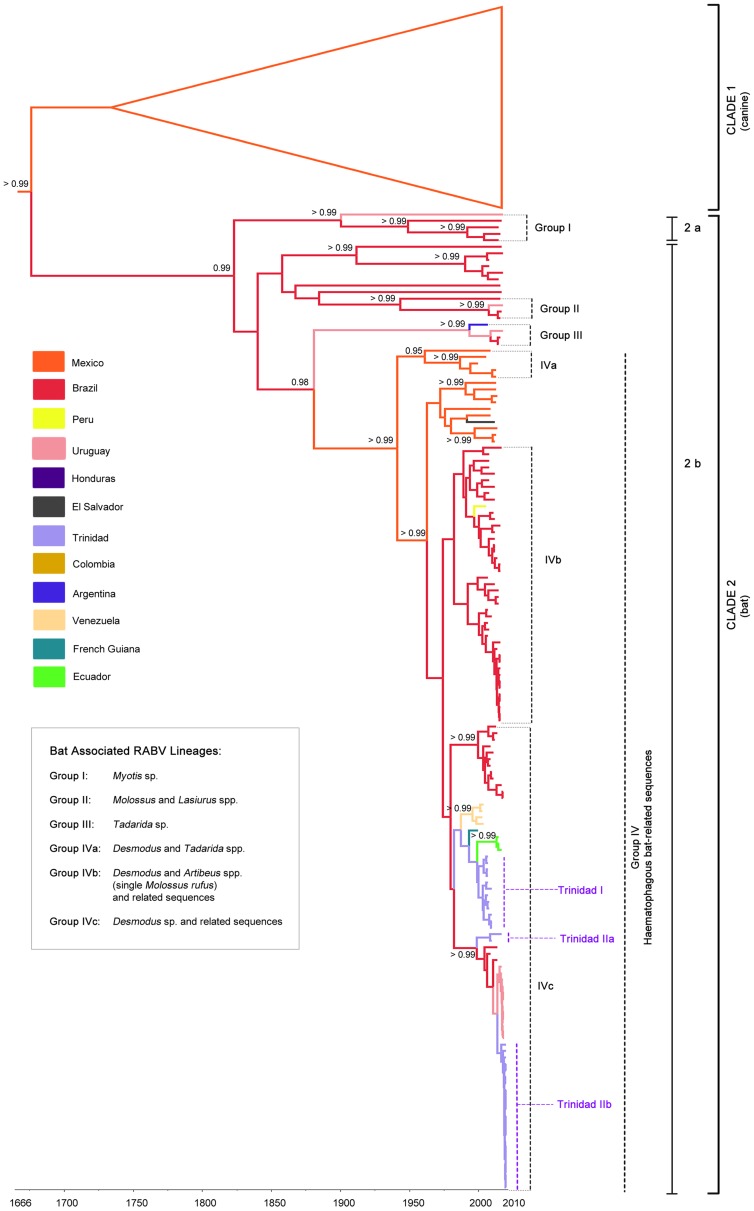
Bayesian Maximum Clade Credibility (MCC) tree inferred for RABV in the Americas based on N gene sequences (363 nt) from South and Central America [including Mexico] and Trinidad. Clade credibilities of 95% and over are indicated in black at the relevant nodes. Terminal branches are coloured according to the sampled location and internal branches are coloured according to the most probable (modal) location of their parental nodes. Major clades and Trinidadian lineages are labeled accordingly. Chiropteran phylogenetic clusters are identified by dotted bar lines to the right and labeled according to group (species) designation which is further described in the inset.

The Bayesian maximum clade credibility (MCC) tree inferred from the complete data set of RABV nucleoprotein gene sequences (from Trinidad and from South and Central America) is divided into two major clades (Figure 2). Clade 1 is comprised of RABV variants associated with canines and Clade 2 consists of bat-associated RABV variants (isolated from rabid bats or other rabid mammals infected by bats) that generally cluster into four well-supported lineages (Groups I–IV; posterior probability = >0.99) defined by the bat species that maintains the variant enzootically (Figure 2 inset).

Within Clade 2 there are two well-defined sub-clades designated 2a and 2b arising from a node with posterior probability of 0.99. Subclade 2a contains RABV sequences associated with *Myotis* sp. bats (Group I) while sub-clade 2b primarily encompasses sequences associated with rabies transmitted by vampire bats, with exception of three independent lineages. The latter being Group II which is associated with RABV in *Lasiurus* and *Molossus* spp. bats from South America, Group III associated with *Tadarida brasiliensis* bats and Group IVa which splits all RABV sequences associated with vampire bats from Central and South America from those of western Mexico and *Tadarida brasiliensis* from North America (represented by 1999_GU991830_TBM_MEX) [38]. Of the remaining predominantly vampire bat (*Desmodus rotundus*) associated lineages within subclade 2b, Group IVb also contains *Desmodus-*related RABV isolates derived from *Artibeus* and *Molossus* spp. bats in Brazil while IVc comprises only sequences derived from *Desmodus* and non-chiropteran species.

All of the Trinidad RABV sequences (n = 37) belong to Group IV within sub-clade 2b suggesting *D. rotundus* as the source of the Trinidad outbreaks. They clustered into two largely temporally defined groups designated Trinidad I and II. Trinidad I includes sequences isolated in 1997, 1998 and 2000 (n = 12) primarily from the northeast region of the island, and is most closely related to RABV from Ecuador (2005, 2007), Venezuela (1993, 1994) and French Guiana (1990). Trinidad II contains all of the 2010 sequences (n = 23) and two earlier sequences (2000, 2007), all from the southwest of the island. The earlier Trinidad sequences grouped together (Trinidad IIa) and were more closely related to Brazilian sequences (1999, 2004). Sequences derived from the 2010 epizootic (Trinidad IIb) clustered with RABV sequences from Uruguay (2007–2008).

### Inference of evolutionary rates, dates of divergence and geographic origins of Trinidadian RABV

**Figure 3:**
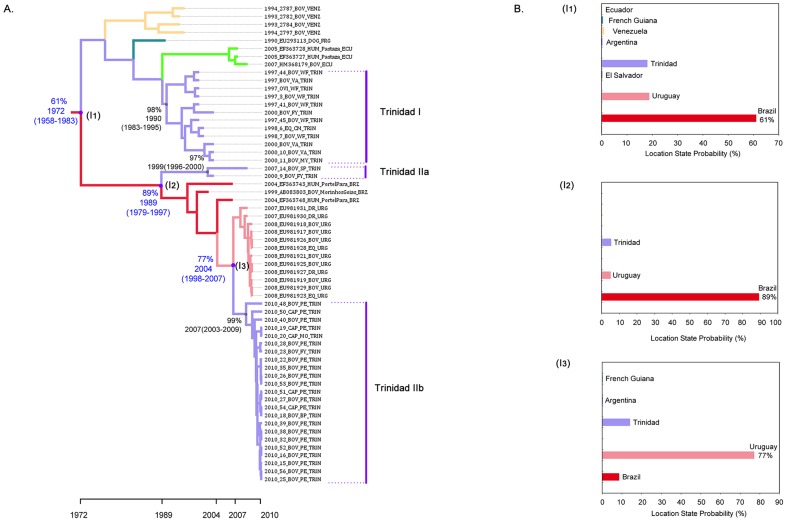
(A) Enlargement of the section of MCC tree containing Trinidadian lineages. The location state probabilities for selected nodes are shown as percentages. The estimated dates of divergence from mainland lineages and mean dates of existence for the most recent common ancestor (MRCA) for lineages containing Trinidadian sequences (with 95% HPD in parentheses) are shown in blue and black respectively to the left of relevant nodes. (**B**) Histogram inserts indicating the location state probabilities for the estimated introductions of RABV that gave rise to Trinidad lineages I (I1), IIa (I2) and IIb (I3).

The phylogeographic analysis supports three temporally separate introductions of RABV from the mainland into Trinidad (Figure 3). Trinidad lineages I and IIa are estimated to be descendants of RABV that most probably existed in Brazil (location state probabilities = 61% and 89%) around 1972 (95% HPD 1958–1983) and 1989 (95% HPD 1979–1997) respectively. In the case of Trinidad IIb (the lineage associated with the 2010 outbreak in Trinidad), the most recent common ancestor is estimated to have descended from an ancestor that existed in Uruguay around 2004 (95% HPD 1998–2007; location state probability = 77%). Lineage expansion after each independent vampire bat-associated rabies introduction is estimated to have occurred within Trinidad (location state probabilities ≥95%) around 1990 (95% HPD 1983–1995) in the case of Lineage I, 1999 (95% HPD 1996–2000) for Lineage IIa, and 2007 (95% HPD 2003–2009) for Lineage IIb with evolutionary rates of 5.24E-4 (95% HPD 1.66E-4 - 9.44E-4), 5.62E-4 (95% HPD 1.94E-4 - 9.91E-4) and 6.24E-4 (95% HPD 2.08E-4 - 1.16E-3) substitutions per site per year respectively. The overall mean rate of evolution estimated for all haematophagus bat-related sequences (Group IV) is 8.16E-4 (95% HPD 2.87E-4 - 1.53E-3) compared with 6.33E-4 (2.22E-4 - 1.16E-3) substitutions per site per year across all of the sampled bat variant lineages (Table S2). The co-existence of both Trinidad I and IIa lineages during the year 2000 indicates a period of co-circulation of distinct RABV variants, specifically in the southwestern village of Fyzabad (see Figure 1).

## Discussion

Trinidadian RABV sequences derived during this study all originated from cases of rabid livestock sampled in Trinidad during the period 1997–2010, including cases from outbreaks in 1997–98, 2000 and 2010. They fall within the bat-associated Clade 2 and cluster into three largely temporally defined lineages designated as Trinidad I, IIa and IIb, with evidence of co-circulation of lineages I and IIa in 2000. These lineages belong to a widespread clade of RABV variants perpetuated by *D. rotundus* (vampire) bats suggesting that the Trinidad outbreaks most likely originate from rabid *D. rotundus* bats. The latter are the main target for rabies surveillance and control activities in Trinidad, on account of their historical and epidemiological association with rabies in both human and domestic animal populations. There were no Trinidadian chiropteran RABV isolates available for analysis in this study but previous evidence suggests that RABV may also be circulating in non-vampire bat species. For example, histopathological examination and animal inoculation conducted in Trinidad have previously identified RABV in frugivorous bats *(Artibeus lituratus*, *Artibeus jamaicensis* and *Carollia perspicillata)* and insectivorous bats *(Diclidurus albus*, *Pteronotus davyi and Molossus major)* [20], [39]. However, as the RABV detected was not genetically characterised, the reservoir host species and source of RABV transmission for these rabid bats remain unknown. Consequently, the role of non-vampire bat populations in the local transmission of the disease remains unclear and warrants further investigation. This is particularly relevant, given that cross-species transmission has been estimated to occur relatively frequently (once for every 73 intra-species transmissions) [40] amongst sympatric bat species, a relationship that has been especially noted between *Artibeus* and *Desmodus* species in Trinidad [20], [41].

Furthermore, studies throughout populated cities in Brazil have shown that *Artibeus* and other bat species such as *Eumops auripendulus*, *Molossus rufus* and *Myotis nigricans* are frequently infected with region-specific *Desmodus* RABV lineages. It is unclear whether these are recurrent spill-over events or whether host shifts may be occurring in one or several of these species [15], [42], [43], [44]. In more extensive surveys [15], fruit bat isolates appear to be sister lineages to RABV isolated from *D. rotundus* and from livestock, which points towards recurrent spill-over as the primary mechanism. However, additional research is needed to clarify the role of other bat species in the maintenance of the vampire bat RABV variant and of specific geographic lineages in nature. Integrated phylogeographic and ecological studies encompassing more comprehensive spatio-temporal samplings of rabid hosts may present a way forward in this regard. Although all of the Trinidadian RABV sequences in this study were found to be vampire bat variant, since there is substantial evidence to suggest that rabies occurs in an enzootic fashion in several other (non-vampire) bat species in the Americas [15], [16], [45], it may also be worth monitoring bat species with migratory habits to determine if RABV variants different from those associated with vampire bats are also being introduced into Trinidad.

In Trinidad, over the period 1971–2010, only one out of 3,868 bats tested (i.e. 0.03%) by DFA tests, under the active vampire bat rabies surveillance program was rabies positive [46]. This surveillance program mainly targets apparently healthy bats from known roosts primarily in the south of the country and is typically guided by public reporting of bat biting cases, so there is some surveillance bias. Nonetheless, this rabies positive rate is comparable to rates of 1% or less reported in natural bat populations elsewhere [47] but is lower than rates of up to 3.3% (n = 12,227) estimated for Trinidad on the basis of histopathological analysis for the presence of Negri bodies in brain tissues during the 1930's [41], [48]. The passive surveillance system in the United States receives more than 20,000 bats (found sick or inside human dwellings) annually for rabies testing, of which close to 6% are confirmed rabid [49]. There is no passive surveillance of comparable scale in Trinidad so it is not possible to compare. However, higher rabies positive rates are expected under passive surveillance assuming that sick and injured bats would account for the majority of bats submitted.

RABV importation into Trinidad was first proposed by early researchers such as Pawan [50] who suggested that the virus was not indigenous to the bat population of Trinidad, and was first introduced around 1925 when the first outbreak of bat-transmitted rabies was documented. It is however likely that bat-associated rabies existed in Trinidad even prior to the 1900's but its presence was masked by the incidence of dog-associated rabies during those times. Nonetheless, the results of our phylogeographic analyses provide statistical support for at least three recent independent introductions of RABV into Trinidad from the mainland (from lineages estimated to have arisen in 1972, 1989 and 2004), with the root state probabilities favoring Brazil and Uruguay as source populations for RABV dissemination to Trinidad. As discussed below, RABV introduction is unlikely to have been direct from these countries but would presumably have occurred via gradual movement of infected bats flying to regions of the mainland neighbouring Trinidad with subsequent entry. The proximity of Trinidad to the Venezuelan coast of South America, (with the closest point being approximately 7 miles from the southwestern peninsula of the island), could easily facilitate this. In fact, the proposed rabies reservoir host (i.e. the *D. rotundus*, bat) has been reported to fly up to 12 miles (20 km) from the roost to the feeding site in a single night [51], which would amply allow for these bats to traverse the short distance from the South American mainland to Trinidad to feed and return or to travel further inland on the island. Finally further supportive evidence for such movement is provided by documented personal accounts of the sighting of bats flying between Trinidad and the mainland [20], [52] and the sudden appearance in Trinidad of a second vampire bat species (*Diaemus youngi*) for the first time in 1954 within *D. rotundus* roosts that had been routinely visited since 1935 [52]. Additionally, phylogenetic similarity between Trinidadian RABV and Venezuelan RABV isolated from northern Venezuelan states has been previously reported [26], [27].

Although there is clear evidence of multiple introductions, Trinidad lineages I and IIa include sequences from viruses sampled several years apart (up to 3 years and 7 years respectively) indicating insular evolution at least for limited periods. Furthermore, based on the inferred locations of origin and the 95% HPD for the ages of the MRCAs of the three Trinidad lineages, the Trinidad lineages are likely to have existed in Trinidad for as many as 14 (lineage I), 4 (lineage IIa) and 7 years (lineage IIb) prior to first being sampled in 1997, 2000 and 2010 respectively.

The overall mean rate estimated across the bat lineages represented in the current study (i.e. 6.33E-4 substitutions per site per year) translates into a relatively recent estimate for the date when the most recent common ancestor for these lineages existed (around 1813; 95% HPD 1716–1895). This is more recent than the estimate for all bat rabies in the Americas reported by Streicker et al (i.e. 1585 [95% HPD 1493–1663]) [53] whose data set was more representative of the bat rabies diversity throughout the Americas having included 21 subspecies, species or genus specific lineages of rabies virus sampled over a period of 37 years (including several lineages from temperate regions of North America). In contrast the data set in the current study was restricted to South and Central America, represented a shorter time frame (13 years) and included a large number of Trinidad sequences, which while appropriate for investigating the origins and evolution of Trinidad RABV cannot not be used as a basis for making more general conclusions about substitution rates and dates of divergence for bat rabies in the wider Americas. Particularly given the over-representation of *D. rotundus* variant (which has a higher mean evolutionary rate than other variants) in our data set and the fact that evolutionary rates for RABV in tropical or subtropical bat species are nearly four times faster than in temperate species [53].

In terms of the mainland origins of the Trinidad lineages, it should be noted that the phylogeographic model used (i.e. the “discrete” model), assumes that at any point along the phylogeny the ancestors for the lineages sampled existed in one of the sampled locations. Thus while Trinidad lineages are clearly descended from Brazilian and Uruguayan lineages, and these countries are the most likely source countries amongst those sampled, since current rabies surveillance systems might not be detecting RABV lineages circulating in large transects of the Amazon region, it is also likely that the introductions occurred via bats infected with RABV lineages originating from undersampled or unsampled countries closer to Trinidad, such as Venezuela or Guyana. There is also the possibility of human-mediated RABV introduction, for example accidental translocation of bats via ships, shipping containers and even aircrafts, which has been documented in previous studies [54]. However, given that the dates of divergence from the Brazilian and Uruguayan ancestors predate lineage expansion within Trinidad by several years, it is more likely that, during this period, RABV from Brazil and Uruguay spread northward from country to country on the mainland until being isolated in Trinidad several years later. Subsequent to our analysis, we received sequence data from two bovine RABV from Guyana (GenBank accession nos: KF424538 and KF424539) which further support the northwards expansion of Lineage IIa from Brazil (data not shown). This limitation of the discrete model in BEAST may be addressed through implementation of the more realistic “continuous trait” model that allows for diffusion over a continuous landscape [55]. However the performance of this model relies on very specific locations of origin (i.e. GIS co-ordinates), which were not available for the sequences used in the present study.

In light of the confirmed vampire bat origin of RABV circulating within Trinidad and statistically supported evidence of repeated importation from the mainland, further studies should be conducted to investigate the relationship between RABV spatiotemporal dynamics and vampire bat population ecology, in particular any movement between the mainland and Trinidad. A better understanding of this relationship would allow for more focused targeting of vampire bat surveillance activities.

Vampire bats are clearly the most significant reservoir for RABV in Trinidad [18], [56]. However, since the last reported case of canine transmitted animal rabies in 1914 [57], other than a mongoose survey in 1954 [58] (the results of which indicated a rabies-free mongoose population [18]), there has been no surveillance targeting wildlife (non-chiropteran) and free-living animals. Therefore, the extent to which other wildlife species (including other bat species) may be involved in RABV transmission in Trinidad remains unknown. In this light, a paradigm shift towards passive surveillance focusing on dead, sick and injured bats and wildlife could contribute to filling this gap.

## Supporting Information

Figure S1 Animal Rabies Cases confirmed at the Veterinary Diagnostic Laboratory in Trinidad by Direct Fluorescent Antibody Testing (DFA) during the period 1997–2010.
(TIF)

Table S1 List of sequences included in the data set from Central and South American RABV isolates (1990–2008) by country, year and source of virus.
(DOCX)

Table S2 Rates of evolution (nucleotide substitution rate per year) for significant clades and lineages.
(DOCX)
