# Supplementary material for: Evolutionary History and Phylogeography of Rabies Viruses Associated with Outbreaks in Trinidad
Source: PLoS Negl Trop Dis. 2013 Aug 22;7(8):e2365. doi: 10.1371/journal.pntd.0002365 (PMC3749974; doi:10.1371/journal.pntd.0002365)
Supplement: Table S1 — List of sequences included in the data set from Central and South American RABV isolates (1990–2008) by country, year and source of virus. (DOCX) [file pntd.0002365.s002.docx]

**Table S1:** List of sequences included in the data set from Central and South American RABV isolates (1990 – 2008), by country,

year and source of virus.

| **Sequence ID** | **Accession Number** | **Country** | **Year** | **Source** | **Clade** | **Bat Group** |
| --- | --- | --- | --- | --- | --- | --- |
| 2008_GQ160959_BOV_BRZ | GQ160959 | Brazil | 2008 | Bovine | 2b | IVc |
| 2008_GQ160957_EQ_BRZ | GQ160957 | Brazil | 2008 | Equine | 2b | IVc |
| 2008_FJ829028_HUM_BRZ | FJ829028 | Brazil | 2008 | Human | 2b | n/a |
| 2008_FJ829027_MAM_BRZ | FJ829027 | Brazil | 2008 | Marmoset | 2b | n/a |
| 2008_EU981921_BOV_URG | EU981921 | Uruguay | 2008 | Bovine | 2b | IVc |
| 2008_EU981926_BOV_URG | EU981926 | Uruguay | 2008 | Bovine | 2b | IVc |
| 2008_EU981919_BOV_URG | EU981919 | Uruguay | 2008 | Bovine | 2b | IVc |
| 2008_EU981918_BOV_URG | EU981918 | Uruguay | 2008 | Bovine | 2b | IVc |
| 2008_EU981929_BOV_URG | EU981929 | Uruguay | 2008 | Bovine | 2b | IVc |
| 2008_EU981925_BOV_URG | EU981925 | Uruguay | 2008 | Bovine | 2b | IVc |
| 2008_EU981917_BOV_URG | EU981917 | Uruguay | 2008 | Bovine | 2b | IVc |
| 2008_EU981927_DR_URG | EU981927 | Uruguay | 2008 | *Desmodus rotundus* | 2b | IVc |
| 2008_EU981928_EQ_URG | EU981928 | Uruguay | 2008 | Equine | 2b | IVc |
| 2008_EU981923_EQ_URG | EU981923 | Uruguay | 2008 | Equine | 2b | IVc |
| 2008_EU981924_MM_URG | EU981924 | Uruguay | 2008 | *Molossus molossus* | 2b | II |
| 2008_EU981922_MYO_URG | EU981922 | Uruguay | 2008 | *Myotis spp* | 2a | I |
| 2008_EU981920_TB_URG | EU981920 | Uruguay | 2008 | *Tadarida brasiliensis* | 2a | III |
| 2007_GQ160919_BOV_BRZ | GQ160919 | Brazil | 2007 | Bovine | 2b | IVc |
| 2007_GQ160914_EQ_BRZ | GQ160914 | Brazil | 2007 | Equine | 2b | IVb |
| 2007_GU552823_LE_BRZ | GU552823 | Brazil | 2007 | *Lasiurus ega* | 2b | II |
| 2007_FJ829024_MAM_BRZ | FJ829024 | Brazil | 2007 | Mamoset | 2b | n/a |
| 2007_GU552796_MM_BRZ | GU552796 | Brazil | 2007 | *Molossus molossus* | 2b | II |
| 2007_GU552816_MN_BRZ | GU552816 | Brazil | 2007 | *Myotis nigricans* | 2a | I |
| 2007_HM368179_BOV_ECU | HM368179 | Ecuador | 2007 | Bovine | 2b | IVc |
| 2007_GU991847_DOG_MEX | GU991847 | Mexico | 2007 | Canine | 1 | n/a |
| 2007_FJ228543_SKU_MEX | FJ228543 | Mexico | 2007 | Skunk | 1 | n/a |
| 2007_EU981931_DR_URG | EU981931 | Uruguay | 2007 | *Desmodus rotundus* | 2b | IVc |
| 2007_EU981930_DR_URG | EU981930 | Uruguay | 2007 | *Desmodus rotundus* | 2b | IVa |
| 2006_GQ915422_BOV_BRZ | GQ915422 | Brazil | 2006 | Bovine | 2b | IVb |
| 2006_GQ915424_BOV_BRZ | GQ915424 | Brazil | 2006 | Bovine | 2b | IVb |
| 2006_GQ915421_BOV_BRZ | GQ915421 | Brazil | 2006 | Bovine | 2b | IVb |
| 2006_GQ915423_BOV_BRZ | GQ915423 | Brazil | 2006 | Bovine | 2b | IVb |
| 2006_GQ915420_BOV_BRZ | GQ915420 | Brazil | 2006 | Bovine | 2b | IVb |
| 2006_GQ915419_BOV_BRZ | GQ915419 | Brazil | 2006 | Bovine | 2b | IVb |
| 2006_EF428578_DR_BRZ | EF428578 | Brazil | 2006 | *Desmodus rotundus* | 2b | IVb |
| 2006_EF428576_DR_BRZ | EF428576 | Brazil | 2006 | *Desmodus rotundus* | 2b | IVb |
| 2006_EF428579_DR_BRZ | EF428579 | Brazil | 2006 | *Desmodus rotundus* | 2b | IVb |
| 2006_EF428580_DR_BRZ | EF428580 | Brazil | 2006 | *Desmodus rotundus* | 2b | IVb |
| 2006_EF428582_DR_BRZ | EF428582 | Brazil | 2006 | *Desmodus rotundus* | 2b | IVb |
| 2006_EF428577_DR_BRZ | EF428577 | Brazil | 2006 | *Desmodus rotundus* | 2b | IVb |
| 2006_EF428581_DR_BRZ | EF428581 | Brazil | 2006 | *Desmodus rotundus* | 2b | IVb |
| 2006_GU204250_DR_BRZ | GU204250 | Brazil | 2006 | *Desmodus rotundus* | 2b | IVb |
| 2006_GQ915416_EQ_BRZ | GQ915416 | Brazil | 2006 | Equine | 2b | IVb |
| 2006_GU552824_LC_BRZ | GU552824 | Brazil | 2006 | *Lasiurus cinerus* | 2b | II |
| 2006_GU552822_LE_BRZ | GU552822 | Brazil | 2006 | *Lasiurus ega* | 2b | II |
| 2006_GU552789_MM_BRZ | GU552789 | Brazil | 2006 | *Molossus molossus* | 2b | II |
| 2006_GU552812_MN_BRZ | GU552812 | Brazil | 2006 | *Myotis nigricans* | 2a | I |
| 2006_GU552786_TB_BRZ | GU552786 | Brazil | 2006 | *Tadarida brasiliensis* | 2b | III |
| 2005_EF194167_DOG_BRZ | EF194167 | Brazil | 2005 | Canine | 1 | n/a |
| 2005_EF152258_HUM_BRZ | EF152258 | Brazil | 2005 | Human | 1 | n/a |
| 2005_EF363739_HUM_TuriacuMaranha_BRZ | EF363739 | Brazil | 2005 | Human | 2b | IVb |
| 2005_EF363757_HUM_ViseuPara_BRZ | EF363757 | Brazil | 2005 | Human | 2b | IVb |
| 2005_EF363749_HUM_AugustoCorreaP_BRZ | EF363749 | Brazil | 2005 | Human | 2b | IVb |
| 2005_FJ829027_MAM_BRZ | FJ829027 | Brazil | 2005 | Mamoset | 2b | n/a |
| 2005_GU552819_MN_BRZ | GU552819 | Brazil | 2005 | *Myotis nigricans* | 2a | I |
| 2005_GU552814_MYO_BRZ | GU552814 | Brazil | 2005 | *Myotis spp* | 2a | I |
| 2005_GU552787_TB_BRZ | GU552787 | Brazil | 2005 | *Tadarida brasiliensis* | 2b | III |
| 2005_EF363728_HUM_Pastaza_ECU | EF363728 | Ecuador | 2005 | Human | 2b | IVc |
| 2005_EF363727_HUM_Pastaza_ECU | EF363727 | Ecuador | 2005 | Human | 2b | IVc |
| 2005_GU991845_SKU_MEX | GU991845 | Mexico | 2005 | Skunk | 1 | n/a |
| 2005_FJ228500_HUM_PERU | FJ228500 | Peru | 2005 | Human | 1 | n/a |
| 2004_GQ915413_BOV_BRZ | GQ915413 | Brazil | 2004 | Bovine | 2b | IVc |
| 2004_GQ915433_EQ_BRZ | GQ915433 | Brazil | 2004 | Equine | 2b | IVb |
| 2004_EF363743_HUM_PortelPara_BRZ | EF363743 | Brazil | 2004 | Human | 2b | IVc |
| 2004_EF363748_HUM_PortelPara_BRZ | EF363748 | Brazil | 2004 | Human | 2b | IVc |
| 2004_GQ915411_OVI_BRZ | GQ915411 | Brazil | 2004 | Ovine | 2b | IVb |
| 2004_EU086161_DOG_COL | EU086161 | Columbia | 2004 | Canine | 1 | n/a |
| 2004_FJ228488_BOV_MEX | FJ228488 | Mexico | 2004 | Bovine | 2b | n/a |
| 2004_GU991825_HUM_MEX | GU991825 | Mexico | 2004 | Human | 2b | n/a |
| 2004_FJ228499_DOG_PERU | FJ228499 | Peru | 2004 | Canine | 1 | n/a |
| 2004_FJ228501_FOX_PERU | FJ228501 | Peru | 2004 | Fox | 1 | n/a |
| 2003_GQ915406_BOV_BRZ | GQ915406 | Brazil | 2003 | Bovine | 2b | IVc |
| 2003_EF152240_CAT_BRZ | EF152240 | Brazil | 2003 | Feline | 1 | n/a |
| 2003_FJ228491_BOV_MEX | FJ228491 | Mexico | 2003 | Bovine | 2b | n/a |
| 2003_FJ228490_BOV_MEX | FJ228490 | Mexico | 2003 | Bovine | 2b | n/a |
| 2003_GU991823_BOV_MEX | GU991823 | Mexico | 2003 | Bovine | 2b | n/a |
| 2003_FJ228493_BOV_MEX | FJ228493 | Mexico | 2003 | Bovine | 2b | IVa |
| 2003_GU991828_BOV_MEX | GU991828 | Mexico | 2003 | Bovine | 2b | IVa |
| 2003_FJ228489_OVI_MEX | FJ228489 | Mexico | 2003 | Ovine | 2b | n/a |
| 2003_GU991844_SKU_MEX | GU991844 | Mexico | 2003 | Skunk | 1 | n/a |
| 2002_AB201802_AL_Sao Paulo_BRZ | AB201802 | Brazil | 2002 | *Artibeus liturasus* | 2b | IVb |
| 2002_AB297627_ART_Rio de Janeiro_BRZ | AB297627 | Brazil | 2002 | *Artibeus spp* | 2b | IVb |
| 2002_GQ915430_BOV_BRZ | GQ915430 | Brazil | 2002 | Bovine | 2b | IVb |
| 2002_GQ915426_BOV_BRZ | GQ915426 | Brazil | 2002 | Bovine | 2b | IVb |
| 2002_GQ915428_BOV_BRZ | GQ915428 | Brazil | 2002 | Bovine | 2b | IVb |
| 2002_GQ915429_BOV_BRZ | GQ915429 | Brazil | 2002 | Bovine | 2b | IVb |
| 2002_GQ915432_BOV_BRZ | GQ915432 | Brazil | 2002 | Bovine | 2b | IVa |
| 2002_GQ915431_EQ_BRZ | GQ915431 | Brazil | 2002 | Equine | 2b | IVb |
| 2002_AB201817_MR_Sao Paulo_BRZ | AB201817 | Brazil | 2002 | *Molossus rufus* | 2a | IVb |
| 2002_GQ915427_OVI_BRZ | GQ915427 | Brazil | 2002 | Ovine | 2b | IVb |
| 2002_FJ228492_HUM_SAL | FJ228492 | El Salvador | 2002 | Human | 2b | n/a |
| 2002_GU991824_BOV_MEX | GU991824 | Mexico | 2002 | Bovine | 2b | n/a |
| 2002_FJ228525_DOG_MEX | FJ228525 | Mexico | 2002 | Canine | 1 | n/a |
| 2002_FJ228527_FEL_MEX | FJ228527 | Mexico | 2002 | Feline | 1 | n/a |
| 2002_FJ228484_SKU_MEX | FJ228484 | Mexico | 2002 | Skunk | 1 | n/a |
| 2001_GQ915425_BOV_BRZ | GQ915425 | Brazil | 2001 | Bovine | 2b | IVb |
| 2001_FJ649172_BOV_BRZ | FJ649172 | Brazil | 2001 | Bovine | 2b | IVc |
| 2001_FJ649186_EQ_BRZ | FJ649186 | Brazil | 2001 | Equine | 2b | IVc |
| 2001_FJ228517_DOG_HON | FJ228517 | Honduras | 2001 | Canine | 1 | n/a |
| 2001_FJ228515_DOG_MEX | FJ228515 | Mexico | 2001 | Canine | 1 | n/a |
| 2001_GU991846_SKU_MEX | GU991846 | Mexico | 2001 | Skunk | 1 | n/a |
| 2000_FJ649156_BOV_BRZ | FJ649156 | Brazil | 2000 | Bovine | 2b | IVc |
| 2000_FJ649184_EQ_BRZ | FJ649184 | Brazil | 2000 | Equine | 2b | IVb |
| 2000_FJ228516_DOG_MEX | FJ228516 | Mexico | 2000 | Canine | 1 | n/a |
| 2000_FJ228533_DOG_MEX | FJ228533 | Mexico | 2000 | Canine | 1 | n/a |
| 2000_FJ228545_SKU_MEX | FJ228545 | Mexico | 2000 | Skunk | 1 | n/a |
| 1999_FJ649103_BOV_BRZ | FJ649103 | Brazil | 1999 | Bovine | 2b | IVc |
| 1999_FJ649104_BOV_BRZ | FJ649104 | Brazil | 1999 | Bovine | 2b | IVc |
| 1999_AB083803_BOV_MorinhosGoias_BRZ | AB083803 | Brazil | 1999 | Bovine | 2b | IVc |
| 1999_FJ649178_EQ_BRZ | FJ649178 | Brazil | 1999 | Equine | 2b | IVc |
| 1999_GU991826_BOV_MEX | GU991826 | Mexico | 1999 | Bovine | 2b | n/a |
| 1999_FJ228485_BOV_MEX | FJ228485 | Mexico | 1999 | Bovine | 1 | n/a |
| 1999_FJ228507_DOG_MEX | FJ228507 | Mexico | 1999 | Canine | 1 | n/a |
| 1999_GU991827_HUM_MEX | GU991827 | Mexico | 1999 | Human | 2b | n/a |
| 1999_GU991830_TBM_MEX | GU991830 | Mexico | 1999 | *Tadarida brasiliensis mexicana* | 2b | IVa |
| 1998_AB117970_AL_BRZ | AB117970 | Brazil | 1998 | *Artibeus liturasus* | 2b | IVb |
| 1998_AB117969_AL_Sao Paulo_BRZ | AB117969 | Brazil | 1998 | *Artibeus liturasus* | 2b | IVb |
| 1998_AB117972_AP_BRZ | AB117972 | Brazil | 1998 | *Artibeus planirostris* | 2b | IVb |
| 1998_FJ649075_BOV_BRZ | FJ649075 | Brazil | 1998 | Bovine | 2b | IVb |
| 1998_FJ649074_BOV_BRZ | FJ649074 | Brazil | 1998 | Bovine | 2b | IVb |
| 1998_FJ649176_EQ_BRZ | FJ649176 | Brazil | 1998 | Equine | 2b | IVb |
| 1998_FJ829025_HUM_BRZ | FJ829025 | Brazil | 1998 | Human | 2b | n/a |
| 1998_FJ829026_MAM_BRZ | FJ829026 | Brazil | 1998 | Mamoset | 2b | n/a |
| 1998_FJ228523_DOG_MEX | FJ228523 | Mexico | 1998 | Canine | 1 | n/a |
| 1997_EU293116_TB_ARG | EU293116 | Argentina | 1997 | *Tadarida brasiliensis* | 2b | III |
| 1997_FJ649057_BOV_BRZ | FJ649057 | Brazil | 1997 | Bovine | 2b | IVb |
| 1997_FJ649056_BOV_BRZ | FJ649056 | Brazil | 1997 | Bovine | 2b | IVb |
| 1997_AF070449_DR_BRZ | AF070449 | Brazil | 1997 | *Desmodus rotundus* | 2b | IVb |
| 1997_FJ649173_EQ_BRZ | FJ649173 | Brazil | 1997 | Equine | 2b | IVb |
| 1996_AY854587_VAM_Morelos_MEX | AY854587 | Mexico | 1996 | Vampire bat | 2b | IVa |
| 1996_AF045166_HUM_PERU | AF045166 | Peru | 1996 | Human | 2b | IVb |
| 1995_FJ228524_DOG_MEX | FJ228524 | Mexico | 1995 | Canine | 1 | n/a |
| 1995_FJ228532_DOG_MEX | FJ228532 | Mexico | 1995 | Canine | 1 | n/a |
| 1994_FJ228522_BOV_MEX | FJ228522 | Mexico | 1994 | Bovine | 1 | n/a |
| 1994_FJ228511_BOV_MEX | FJ228511 | Mexico | 1994 | Bovine | 1 | n/a |
| 1994_2787_BOV_VENZ | 2787**^¥^** | Venezuela | 1994 | Bovine | 2b | IVc |
| 1994_2797_BOV_VENZ | 2797**^¥^** | Venezuela | 1994 | Bovine | 2b | IVc |
| 1993_2784_BOV_VENZ | 2784**^¥^** | Venezuela | 1993 | Bovine | 2b | IVc |
| 1993_2782_BOV_VENZ | 2782**^¥^** | Venezuela | 1993 | Bovine | 2b | IVc |
| 1991_FJ228531_CAP_MEX | FJ228531 | Mexico | 1991 | Caprine | 1 | n/a |
| 1991_FJ228521_DOG_MEX | FJ228521 | Mexico | 1991 | Canine | 1 | n/a |
| 1991_FJ228514_DOK_MEX | FJ228514 | Mexico | 1991 | Donkey | 1 | n/a |
| 1991_FJ228510_POR_MEX | FJ228510 | Mexico | 1991 | Porcine | 1 | n/a |
| 1990_EU293113_DOG_GUY | EU293113 | French Guiana | 1990 | Canine | 2b | IVa |
| 1990 _AY877433_BOV_Chiapas_MEX | AY877433 | Mexico | 1990 | Bovine | 2a | IVa |
| 1990_FJ228519_FEL_MEX | FJ228519 | Mexico | 1990 | Feline | 1 | n/a |

**^¥^** Sequences obtained from the repository of the Rabies Program, CDC and the Wildlife Zoonoses and Vector-borne Diseases Research Group , Animal Health & Veterinary Laboratories Agency (Weybridge)
