# Supplementary material for: Evolutionary History and Phylogeography of Rabies Viruses Associated with Outbreaks in Trinidad
Source: PLoS Negl Trop Dis. 2013 Aug 22;7(8):e2365. doi: 10.1371/journal.pntd.0002365 (PMC3749974; doi:10.1371/journal.pntd.0002365)
Supplement: Table S2 — Rates of evolution (nucleotide substitution rate per year) for significant clades and lineages. (DOCX) [file pntd.0002365.s003.docx]

**Table S2:** Rates of evolution (nucleotide substitution rate per year) for significant clades and lineages

| **Lineage** | **n** | **Rate**  **substitutions/ site/ year (95% HPD)** |
| --- | --- | --- |
| RABV (South and Central America) | 183 | 6.11E-4 (3.56E-4 - 8.85E-4) |
| Canine variant | 32 | 5.89E-4 (1.50E-4 - 1.05E-3) |
| Bat variant | 151 | 6.33E-4 (2.22E-4 - 1.16E-3) |
| Trinidad I | 12 | 5.24E-4 (1.66E-4 - 9.44E-4) |
| Trinidad IIa | 2 | 5.62E-4 (1.94E-4 - 9.91E-4) |
| Trinidad IIb | 23 | 6.24E-4 (2.08E-4 - 1.16E-3) |
| Group I (*Myotis* spp) | 5 | 5.97E-4 (2.32E-4 - 1.08E-3) |
| Group II (*Lasiurus, Molossus* spp) | 4 | 7.41E-4 (2.66E-4 - 1.37E-3) |
| Group III (*Tadarida braziliensis* spp) | 4 | 4.35E-4 (1.81E-4 - 7.29E-4) |
| Group IV (*Desmodus rotundus*) | 138 | 8.16E-4 (2.87E-4 - 1.53E-3) |
